# Progress in Occupational Asthma

**DOI:** 10.3390/ijerph17124553

**Published:** 2020-06-24

**Authors:** Angelica I. Tiotiu, Silviya Novakova, Marina Labor, Alexander Emelyanov, Stefan Mihaicuta, Plamena Novakova, Denislava Nedeva

**Affiliations:** 1 Department of Pulmonology, University Hospital of Nancy, 54000 Nancy, France; 2 Development, Adaptation and Disadvantage, Cardiorespiratory Regulations and Motor Control (EA 3450 DevAH), University of Lorraine, 54000 Nancy, France; 3 Allergy Unit, Internal Consulting Department, University Hospital “St. George”, 4000 Plovdiv, Bulgaria; novakova66@yahoo.com; 4 Department of Pulmonology, University Hospital Centre Osijek, 31000 Osijek, Croatia; marina.os21@gmail.com; 5 Medical Faculty Osijek, J.J. Strossmayer University, 31000 Osijek, Croatia; 6 Department of Respiratory Medicine, North-Western Medical University, 191015 Saint-Petersburg, Russia; emelav@inbox.ru; 7 Victor Babes University of Medicine and Pharmacy, 300120 Timisoara, Romania; 8 Clinic of Clinical Allergy, Medical University, 1000 Sofia, Bulgaria; nplamena@yahoo.com; 9 Medical University Sofia, 1000 Sofia, Bulgaria; denislava.nedeva@gmail.com

**Keywords:** occupational asthma, phenotypes, diagnosis, treatment

## Abstract

Occupational asthma (OA) represents one of the major public health problems due to its high prevalence, important social and economic burden. The aim of this review is to summarize current data about clinical phenotypes, biomarkers, diagnosis and management of OA, a subtype of work-related asthma. Most studies have identified two phenotypes of OA. One is sensitizer-induced asthma, occuring after a latency period and caused by hypersensitivity to high- or low-molecular weight agents. The other is irritant-induced asthma, which can occur after one or more exposures to high concentrations of irritants without latency period. More than 400 agents causing OA have been identified and its list is growing fast. The best diagnostic approach for OA is a combination of clinical history and objective tests. An important tool is a specific inhalation challenge. Additional tests include assessments of bronchial hyperresponsiveness to methacholine/histamine in patients without airflow limitations, monitoring peak expiratory flow at- and off-work, sputum eosinophil count, exhaled nitric oxide measurement, skin prick tests with occupational allergens and serum specific IgE. Treatment of OA implies avoidance of exposure, pharmacotherapy and education. OA is a heterogeneous disease. Mechanisms of its different phenotypes, their diagnosis, role of new biomarkers and treatment require further investigation.

## 1. Introduction

Asthma is a heterogeneous disease characterized by a chronic airway inflammation and defined by recurring episodes of wheezing, shortness of breath, chest tightness and coughing that vary over time and in intensity, associated with variable expiratory flow limitation [1]. Approximately 360 million people worldwide are affected and it is thought that up to 25% of adult-onset asthma is work-related [2,3]. 

Work-related asthma is the term used to define asthma worsened by the workplace and encompasses both occupational asthma (OA) and work-exacerbated asthma [3,4,5,6]. OA is de novo asthma induced by either sensitization to a specific substance or a chemical at work, which is termed sensitizer-induced OA (SI-OA) or by exposure to high concentrations of an inhaled irritant found in the workplace, which is termed irritant-induced OA (II-OA) [3,4,5,7]. Sensitizing agents that cause OA are classified into high-molecular weight (HMW) (glyco) proteins (>10 kD) from vegetable or animal origins and low-molecular-weight (LMW) agents (<1 kD) which include chemicals, metals and wood dust [8]. If SI-OA has a latency period of exposure and sensitization before the symptoms begin, II-OA includes both an acute onset form, the “reactive airways dysfunction syndrome” (RADS) where asthma symptoms start 24 h after a single high level exposure to an inhaled irritant and delayed onset forms with latency where asthma develops insidiously over time after either an acute exposure (e.g., World Trade Center rescue workers) or after repeated lower level exposures (e.g., professional cleaners), also called “low-dose RADS” [3,7].Work-exacerbated asthma implies a preexisting or concurrent asthma that is worsened by exposure to non-specific stimuli at the work, but not caused by it [3,4].

More than 400 agents causing OA have been identified, new causative agents are reported each year so the list is growing fast but is rapidly outdated and perpetually incomplete [6,7]. The diagnosis of OA is difficult, requiring confirmation for the diagnosis of asthma, plus evidence that the asthma was caused by workplace conditions [7]. An accurate diagnosis of OA is a very important viewing of the significant health consequences for affected workers, but also the substantial socio-economic impact [3,5,7].

The aim of this review is to summarize current data about clinical phenotypes, biomarkers, diagnosis and management strategies of OA which represents a major public health problem due to its high prevalence and its important financial-societal burden. 

## 2. Sensitizer-Induced Occupational Asthma (SI-OA)

The most common form of OA is SI-OA, which represents >90% of cases [3]. OA incidence varies between countries (from 13 new cases per million workers in Quebec (Canada) to 178 new cases per million workers in the United States) and industries (e.g., 0.9 cases per 100 person-years for the workers in toluene diisocyanate production, 1.8 cases per 100 person-years for health-care workers using latex gloves and 4.1 cases per 100 person-years among workers exposed to wheat flour) [3]. 

**Figure 1:**
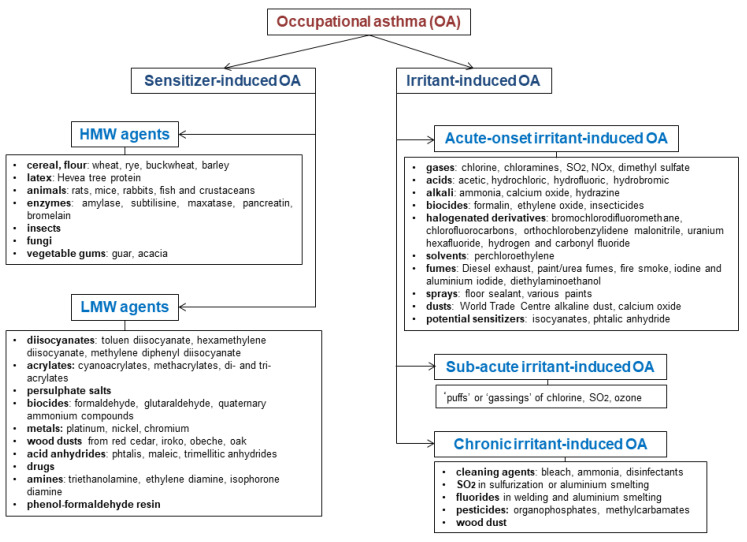
Classification of occupational asthma and the most frequent causal agents; HMW: high-molecular weight; LMW: low-molecular weight.

Efforts to identify risk factors to develop OA have been done. The level of exposure to a sensitizing agent is the most recognized environmental risk factor for OA but evidences suggest that occupational exposure to vapors, dust, gas and fumes increases prevalence of asthma [3,9]. Previous data showed that cigarette smoking increases IgE sensitization to HMW and LMW agents, so smoking could also play a role in the development of OA [6]. Several host factors have been associated with OA. Atopy is a strong risk factor for OA due to HMW agents (e.g., bakers/pastry makers, laboratory animals workers) [3,6,10]. Despite the fact that atopy is associated with a high risk for OA in bakers/pastry-makers (OR = 10.07 95%CI [2.76–36.65]) and hairdressers (OR = 4.94 95%CI [0.66–36.75]) [11], the atopic status seems to not precipitate the occurrence of respiratory symptoms in these specific populations [12]. Other evidence suggests that genetic factors including HLA class II polymorphisms contribute to individual susceptibility for OA induced by LMW agents [3,6,10]. Despite that currently the utility of these factors in practice to determine the ability of a worker to do a job with a risk of sensitization is limited [13], the identification of workers at risk according to their individual characteristics and worker’s education about the workplaces with high levels of occupational allergens/irritants represent very important steps in the prevention of OA [6] Most of HMW and a limited number of LMW agents (e.g., reactive dyes, platinum salts, obeche wood) induce asthma through immunoglobulin E (IgE)-dependent mechanism, while the immunologic pathways involved into the sensitization to LMW agents (e.g., diisocyanates, persulphate salts, aldehydes, anhydride acids, acrylates) are poorly understood [3,5]. HMW agents act as complete antigens and induce the production of specific IgE antibodies, whereas LMW agents act as haptens and bind proteins to form functional antigens. After IgE-cross-linking by the antigen, mast cells release histamine, prostaglandins and cysteinyl-leukotriens. After antigen presentation by dendritic cells, T-lymphocytes differentiate into several subtypes of effector cells. Antigen-activated CD4+ cells can differentiate into Th1-cells which produce interferon-γ and interleukin-2 involved in classical macrophage activation and T lymphocytes differentiation, respectively Th2-cells which release interleukin-4, -5, and -13, activate B-cells, promote IgE-synthesis, recruitment of mast cells, and eosinophilia. Evidence suggests that several LMW agents, such as diisocyanates, can induce innate immune responses by up-regulation/activation of the immune pattern-recognition receptor of monocytes, increasing expression of chemokines that regulate monocyte/macrophage trafficking (e.g., macrophage migration inhibitory factor and monocyte chemoattractant protein 1) and the release of important proinflammatory cytokines such as interleukin-1 or interleukin-15. Innate natural killer cells stimulated by interleukin-15 could also release interleukin-13. Even than all the mechanisms involved are not identified, this data suggests a complex collaboration between innate and adaptive immunes systems in the SI-OA pathogenesis [6]. Figure 1 summarizes the most frequent causal agents for each phenotype of OA.

### 2.1. OA-Induced by HMW Sensitizing Agents

All proteins of animal and plant origin can basically cause an immunologic sensitization and through Th2 pathway induction of IgE production. They all fall into the category of HMW agents [14]. The most common are cereals and flours (wheat, rye, buckwheat, barley), animals (farms, laboratory and seafood), latex and enzymes (amylase, subtilisine, maxatase, pancreatin, bromelain) [3].

Cereals and flours are the oldest reported occupational agents most often seen affecting bakers who are, aside of flours exposed also to some enzymes and storage mites. Wheat is the most frequently encountered cereal, but soya is highly allergenic and thus often the causative agent [15,16]. Exposure to laboratory animals (rats, mice, rabits) causes rapid development of allergy with a short latency period with the strongest source of sensitization being proteins excreted in animal urine [16]. Even bigger animals as cows or horses can also be source of agents triggering OA in farmers or veterinarians [6]. Some fish and crustaceans, especially crab, cause sensitization and OA both in fisherman as well as in people who prepare this type of food. Hevea tree protein from latex gloves caused an outbreak of allergic reactions, particularly in healthcare workers in 1980s, yet today it is much less present due to the availability of alternatives [16]. Enzymes derived from animals as well as from plants used in chemical industry like in detergent/soap production, but also in food and pharmaceutical industries could cause OA [3,15,16]. Some ubiquitous antigens if present in higher concentrations at particular jobs (dust mites for maids in hotels) can also be considered occupational allergens [16].

Previous data have suggested a dose-response relationship between the level of exposure and development of IgE-mediated OA, which could be more evident in an early career. If lower doses of antigen induces reactions only in atopic individuals, exposure to higher concentrations can cause sensitization even in non-atopic persons [3]. This led to the recommendation of 0.2 μg/m^3^ concentration as a threshold value for antigen exposure in Europe. Patients with HMW agents induced OA are usually older individuals and more often active smokers [17].

The classic presentation of SI-OA is a worker who develops asthma symptoms that are worse at work and better on weekends or vacations away from work [7]. A variable latency period ranging from weeks to years after the first exposure to the sensitizer is observed before the onset of respiratory symptoms [6]. A recent study showed a frequent association between OA induced by HMW agents and work-related allergic rhinitis (OR 4.79 [95% CI 3.28–7.12]), conjunctivitis (OR 2.13 [95% CI 1.52–2.98]) and the presence of atopy (OR 1.49 [95% CI 1.09–2.05]) [8].

When performing a specific inhalation challenge (SIC) the reaction to HMW agents is positive after a shorter exposure time and requires higher doses of rescue medications but the bronchial hyperresponsiveness (BHR) detected after SIC is milder than after exposure to LMWA. That by itself confirms that the mechanism of the reaction to HMW and LMW agents is different. The reaction to HMW allergens is mostly driven by IgE antibodies and Th2 response causing the fast release of histamine and hence the early response after short exposure. For that reason HMW agents induce early (80% of them) or dual asthmatic reaction [18]. Quick resolution of histamine is the reason for faster recovery, leaving less damage to respiratory epithelium and thus not causing BHR. Due to this difference in the pathogenesis, the response to controller medication as well as the long term prognosis is different according to the causative agent [19]. These patients have milder course of the disease and often can continue working [17]. When performing work up one needs to bear in mind the difference in the mechanisms of reaction to both agents.

Exhaled nitric oxide level (FeNO) is usually higher after SIC in patients exposed to HMW agents than in patients exposed to LMW agents [20]. Sputum eosinophils percent after SIC correlates with FeNO levels in patients with OA but it is significantly increased in patients exposed to HMW compared to LMW agents. Blood eosinophilia and airflow limitation are also more frequently found in patients with OA induced by HMW agents [8].

### 2.2. OA-Induced by LMW Sensitizing Agents

The LMW compounds are usually small, very reactive chemical molecules that are present in various work settings and industries. In contrast to the HMW agents, the mechanism of sensitization to LMW substances remains poorly understood [8]. LMW agents act as haptens and must conjugate with host proteins (e.g., albumin, hemoglobin) to form new allergenic proteins that can lead to IgE sensitization response [3,5,7]. Since no serum-specific IgE (sIgE) can be detected for most of the LMW agents, alternative pathogenic mechanisms are probably involved such as innate immune responses, epithelial injury, remodeling of the airway wall, oxidative stress and neurogenic inflammation [8,21]. In practice, sIgE could be measured only for a few LMW chemicals—platinum salts, reactive dyes, acid anhydrides, and obeche wood [3,5].

One of the most common LMW agents causing OA are diisocyanates, acrylates, persulfates, acid anhydrides, metals, wood dusts (red cedar, iroko, obeche, oak), biocides (formaldehyde, glutaraldehyde, quaternary ammonium compounds), amines (triethanolamine, ethylene diamine, isophore diamine) and some pharmaceuticals [3,6].

The first case of asthma caused by diisocyanates was reported by Fuchs and Valade in 1951, soon after their introduction in industry [22]. Since then, diisocyantes have become one of the most common causes of OA all over the world. Toluene diisocyanate (TDI), hexamethylene diisocyanate (HDI), diphenylmethane diisocyanate (MDI), isophorone diisocyanate (IPDI) and naphthalene diisocyanate (NDI) are the most used diisocyanates. The workers at risk of exposure are makers of rigid or flexible polyurethane foam, installers of polyurethane foam insulation, urethane spray painters, and those who work with urethane adhesives or urethane molds in foundries [3,6]. Serum-specific IgE can be detected in up to 55% of workers with confirmed OA to diisocyanates [3,23].

Acrylates are monomers that polymerize, and form plastics used in industry as adhesive resins, surface coatings, synthetic textiles, printing ink and hard plastic. In medicine they are used in dentistry and orthopedics. Dental professionals were one of the most affected occupations, but recently cases with OA caused by acrylates were reported especially in beauty industry (e.g., methacrylate for the sculptured nails, cyanoacrylate for the eyelash extension glue) and for optical laboratory technicians (e.g., methacrylate contained by eyeglasses) [24,25,26]. Usually, they cause respiratory symptoms and contact dermatitis, but rhinitis could be also present [25,26]. Most patients have a late or dual asthmatic reaction on SIC, with an increase in FeNO levels after [24,25,26]. The exposure to acrylates in the beauty industry continues to increase, therefore more and more cases of OA and contact dermatitis could be expected in the future.

The sensitization to persulfate solutions is frequent among hairdressers population. The most common symptoms are asthma, coughing, nasal congestion and rhinitis [27] but contact urticaria is also often described [28]. The most important identified allergens are persulfates (ammonium and potassium) and paraphenylenediamine [29]. The sensitization to alkaline persulfates could be proved by skin prick tests (SPT) [28] and SIC is characterized by a late reaction, followed by the increase of FeNO and sputum eosinophils number [30].

Acid anhydrides (phthalic, trimellitic and maleic anhydrides) are used in a variety of chemical processes, mainly in the production of epoxy and alkyd resins used to manufacture a variety of coating materials. They have direct irritant effects, but also can act as sensitizers, leading to the development of OA. Trimellitic anhydride is a very good model of a LMW agent that causes OA through an IgE antibody—mediated mechanism that can be demonstrated by skin prick testing or serologically [30].

Various metals such as chromium, nickel, cobalt, iron, zinc can induce OA in metal-plating workers and welders of stainless steel, but the most frequent cause is the sensitization to platinum salts in metal refinery industry, manufacture of catalysts or cytotoxic drugs [31,32,33,34,35]. IgE-mediated mechanism has been suggested for most of these metals only for platinum salts sIgE could be detected [32,33,34]. The SIC is usually necessary for the confirmation of OA [35]. The exposure to metals seems to be associated with a neutrophilic inflammation and the symptoms of asthma could persist for a long period after the removal from the workplace [31].

Wood dust is a common cause for OA and workers at risk are carpenters, forestry and sawmill workers, furniture and cabinet makers [3,6]. Wood dusts contain LMW sensitizers, such as plicatic acid in red-cedar dust, but can also promote the sensitization by the production of specific IgE antibodies to HMW components (e.g., in olive, pine, chengal, cedrorana, and cabreuva wood) [6]. Current data suggests that plicatic acid can produce lytic damage to bronchial epithelial cells and cause histamine release from bronchial mast cells in workers with Western red cedar asthma [36,37].

Many various cases of OA were reported amongst the workers in the pharmaceutical industry. The most frequent causes for OA in antibiotic manufacturing workers are penicillin’s family compounds (e.g., amoxicillin, ampicillin, piperacillin) and different cephalosporins with sIgE, skin tests and SIC occasionally positive [38]. Cases are also reported for macrolides (spiramicyn, proved by positive SPT and SIC; erythromycin with positive late asthmatic reaction to SIC), tetracycline, thiamphenicol, vancomycin, and colistin (early asthmatic reaction following SIC) [38,39,40]. Case-reports are also published with OA induced by tafenoquine, an antimalarial drug, confirmed by a dual asthmatic reaction following the SIC [41] and ranitidine, an anti-acid medication, with a late asthmatic response after SIC [42]. Even drugs derived from plants like as psyllium, a laxative from *Plantago ovata* or escin, an active ingredient with anti-inflammatory and venotonic properties found in the horse chestnut, induced OA documented by positive SIC [43,44]. In addition, sIgE to psyllium was positive [43]. 

Among biocides, *ortho*-phthalaldehyde (OPA) is an aromatic dialdehyde that has largely replaced glutaraldehyde as a new high-level disinfectant for heat-sensitive medical devices, including endoscopes. OA-induced by OPA was documented by a positive SIC with a delayed asthmatic response (43% fall in FEV1 4 h after exposure) in a worker employed in endoscopic unit [45]. A few cases of OA were also reported with peracetic acid-hydrogen peroxide mixture, an alternative of aldehydes used as disinfectants for endoscopes in hospitals [46,47]. Triclosan is chlorinated diphenyl ether with antimicrobial and antifungal properties used as a biocide in consumer products, such as household cleaners, shampoos, deodorants, and toothpaste. OA induced by triclosan was documented by positive SIC [46]. A recent review evaluated the association between occupational exposure to quaternary ammonium compounds and OA and concluded that currently it is difficult to define this link in raison of the limited data in the literature, studies realized in specific populations (e.g., farmers or cleaners), poor understood pathogenesis and the presence of many confounders (other chemical and biological agents) in the workplace as possible asthmagens [48].

Various cases of OA induced by dyes used in the textile industry were reported. Sudan red is a LMW azoic agent used in industry for coloring of fats, oils, and waxes (including the waxes used in turpentine-based polishes) but also in the production of hair dyes and some temporary tattoos. The SIC is mandatory for the diagnosis and, if positive, could be associated with increase in sputum eosinophils count in the first 24 h after the exposure [49].

Derived from the resin of pines, colophony is a common cause of OA, contact dermatitis and rhinitis. At room temperature resin is brittle, but it melts at stove-top temperature. The professionals at risk for OA induced by colophony are people exposed to solder flux fumes, working in the adhesives, paper and tyre industries, beauticians and welders. OA is usually caused by the exposure of fumes from heated colophony and cases by unheated colophony dust are very rare. The SIC is necessary for the diagnosis of OA induced by colophony [50].

Here we discussed just a few causes of OA induces by LMW but the list is very extensive and new sensitizer agents are described every year. However, several characteristics were identified for this phenotype of SI-OA. Patients with OA due to LMW agents describe more frequently chest tightness at work (OR 2.22, 95%CI [1.59–3.03]), have daily sputum (OR 1.69, 95%CI [1.19–2.38]) and high risk for severe exacerbations (OR 1.32, 95%CI [1.01–1.69]) [8]. According the type of exposure, the risk of severe exacerbations is variable with a prevalence ratio at 3.11 (95%CI 1.56–6.20) for isocyanates, 2.50 (95%CI 1.02–6.14) for epoxy resins, and 1.93 (95%CI 1.09–3.43) for other highly reactive agents such as amines, aldehydes, acids, anhydrides, chromates, curing agents, reactive gases, dyes [51]. LMW agents more frequently provoke late or dual asthmatic responses on SIC and higher increases in BHR [8,19]. Recent data suggest LMW agents is associated with more severe asthma than when induced by HMW agents [18].

## 3. Irritant-Induced Occupational Asthma (II-OA)

The prevalence of II-OA is estimated at 5–10% of cases of OA [52]. According to current guidelines [53] three clinical phenotypes of II-OA are described: (i) definite IIA (RADS or “acute-onset II-OA”) characterized by the rapid onset of asthma within a few hours after a single exposure to very high levels of irritant substances; (ii) probable IIA (“sub-acute II-OA”) defined as insidious asthma developed in workers with multiple symptomatic moderate-high-level exposures to irritants; and (iii) possible IIA (“chronic II-OA“) described as asthma occurring with a delayed-onset after chronic exposure to low-moderate levels of irritants.

The diagnostic criteria for RADS firstly established by Brooks [54] were adapted and include now: the occurrence of asthma symptoms within minutes to hours <24 h following a single identifiable high level exposure to an irritant, the absence of preexisting asthma symptomatology, the exclusion of other pulmonary disorders that can explain the symptoms, and the evidence of BHR or reversible airflow obstruction on spirometry [53]. A large study showed that workers who reported an acute symptomatic inhalation event such as fire, mixing cleaning products, or chemical spills have a three-fold increased risk of new-onset asthma [55]. The most common agents recognized as causes of RADS are gases (chlorine, chloramines, sulfur dioxide, nitrogen oxides, dimethyl sulfate), acids (acetic, hydrochloric, hydrofluoric, hydrobromic), alkali (ammonia, calcium oxide, hydrazine), biocides (formalin, ethylene oxide, fumigating agents, insecticides), halogenated derivatives (bromochlorodifluoromethane, trifluoromethane, chlorofluorocarbons, *ortho*-chlorobenzylidene malonitrile, uranium hexafluoride, hydrogen and carbonyl fluoride), solvents (perchloroethylene), fumes (diesel exhaust, paint fumes, urea fumes, fire smoke, fumes of iodine and aluminum iodide, diethylaminoethanol), sprays (paints, floor sealant), and potential sensitizers like as diisocyanates and phthalic anhydride [7,53]. Several case series are described after a massive exposure to a spill of acetic acid in a hospital [56], to metam sodium pesticide released after the derailment of a train [57], or to a complex mixture of alkaline dust and combustion products following the World Trade Center attacks in 2001 [58,59]. Usually the respiratory symptoms are severe enough to require hospitalization or at least acute medical care [53]. Sometimes, RADS could be associated with “acute irritant-induced rhinitis” [60]. BHR could persist up to 12 years after the event [61].

When asthma occurs with a less acute-onset in a context of multiple moderate-high-level exposure to various irritant compounds (e.g., chlorine, SO_2_, and ozone) the phenotype is defined as “sub-acute II-OA” [53]. For example, a study showed that up to 5% of screened subjects following exposure to liquid chlorine released after a train’s derailment developed asthma 10 months after the accident [62]. Cases are reported in pulp mill workers who had a history of multiple “gassing” episodes that occurred over a period of years, but also in World Trade Centre rescue workers [3,53,63,64]. The causal relationship between the asthma onset and the exposure can be supported in this case by the documentation of repeated symptomatic inhalation accidents requiring medical care [53].

Repeated and/or chronic low-moderate-level exposures to irritants at work are associated with “chronic II-OA” [53]. Currently, this phenotype is reported in workers exposed to cleaning agents who develop insidiously asthma symptoms after a latency period [65]. Although the precise causal factor and the pathogenesis have not been clarified, the frequent use of chlorine bleach, ammonia products, and degreasing sprays has been consistently associated with asthma in workers exposed to cleaning agents [53,65]. The criteria supporting this diagnosis are: (i) adult-onset of asthma or reactivation of previously quiescent asthma; (ii) chronic exposure to irritants; and (iii) absence of an identified sensitizer in the subject’s working environment [53]. Sometimes it is difficult to differentiate this phenotype of OA from work-exacerbated asthma [3,53].

Mechanisms of II-OA are poorly understood. Inhalation of irritants can induce bronchial epithelial damage, resulting in proinflammatory responses, neurogenic inflammation due to exposed nerve endings, increased lung permeability, and remodeling of the airway epithelium [53]. Oxidative stress is one of the mechanisms causing the epithelial damage. Inhalation of irritants induces the release of reactive oxygen species and alarmins. Secreted by stimulated epithelial cells, leukocytes and necrotic cells, alarmins promote the activation of innate immune cells and recruitment/activation of antigen-presenting cells involved in tissue repair and host defense through Toll-like receptors [6]. Chemical irritants could also directly activate sensory nerves by stimulating transient receptor potential (TRP) channels followed by the release of neuropeptides such as substance P, neurokinin A and calcitonin-gene-related peptide triggering aneurogenic inflammation with plasma protein extravasation, vasodilatation, bronchoconstriction, and increased mucus secretion [6,66]. Inhaled irritants could also activate TRP channels expressed by epithelial and inflammatory cells in the airways promoting local inflammation. TRPA1 seems to be a major irritant detector because it could be activated by acrolein, tear gas, vehicle exhaust, nicotine, ozone, hydrogen peroxide and hypochlorite [6]. Bronchial biopsies from patients with II-OA have revealed marked epithelial desquamation, inflammatory changes with predominance of lymphocytes, airway remodeling, and collagen deposition in the bronchial wall [67,68,69]. Despite similar histological changes in II-OA and SI-OA, the basement membrane thickness (sub-epithelial fibrosis) seems to be of greater extent in II-OA than in SI-OA [67,68].

Several factors can influence the airway response to irritant exposure such as chemical properties of the agent (e.g., solubility, reactivity, size of the particles, gas pressure), intensity of exposure, adequacy of environmental ventilation, use of protective equipment and genetic susceptibility [3]. Even though risk factors for II-OA are still unknown, evidence suggests that the high-intensity of exposure is an important predictor factor of the development of II-OA while no association was found for the atopy and smoking [3,64].

## 4. Diagnosis of Occupational Asthma

OA doesn’t only have health, but also social and legal consequences for the workers, their families and employers. It could lead to loss of a job or income and even moving to another town [70]. Therefore, an early diagnosis is a necessity.

**Figure 2:**
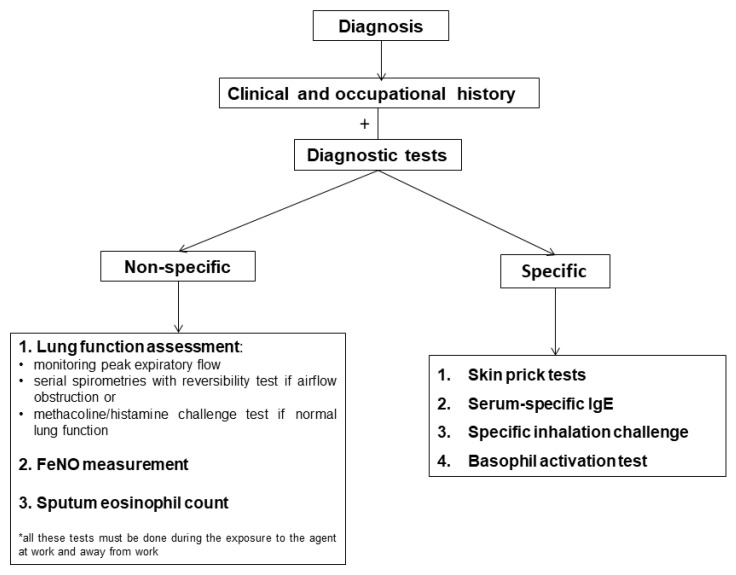
Diagnostic tests in occupational asthma.

The best diagnostic approach for OA is to combine a detailed clinical history with objective diagnostic tests. The latter include evidence for work-related changes in the airways—peak expiratory flow (PEF), non-specific BHR, sputum eosinophil count, FeNO and/or evidence for specific sensitization—SPT, sIgE, SIC, and basophil activation test (BAT) [2]. In the differential diagnosis, it is important to consider other conditions that can mimics asthma symptoms following exposure to irritants in the workplace such as vocal cord dysfunction, hyperventilation syndrome and mass psychogenic illnesses [3,5]. Figure 2 summarizes the possible tests to use in practice for the confirmation of OA diagnosis.

### 4.1. Clinical and Occupational History

The typical history of OA is the appearance or worsening of asthma symptoms at work and their improvement outside the work environment. However, asthma symptoms could be present outside the workplace as late asthmatic reactions or triggered by non-specific stimuli like cold air, fumes or exercise. The remission of symptoms in the evening or during weekends tends to disappear when affected workers continue to be exposed to the sensitizing agent [5]. For these reasons, patients may not aware of the relationship between their occupation and their symptoms, and the diagnosis of OA is usually made 2–4 years following the onset of symptoms [3]. The most relevant items that should be addressed when taking the clinical history include: occupation (description of tasks and processes, work environment, respiratory protective precautions, identification of direct and indirect exposure to potential workplace asthmagens by examining the Material Safety Data Sheets [MSDS]), respiratory symptoms (nature, latency period, and temporal relationship with work exposure), and associated work-related rhinitis/conjunctivitis, urticaria or contact dermatitis [3,5].

Questionnaires are being used for individual assessments and in most epidemiological surveys [70]. Although sensitive, it has been found that questionnaires were not a specific diagnostic tool because the positive predictive value is at 63% while the negative predictive value is at 83% [71]. Despite a high sensitivity of the clinical history for the diagnosis of OA, the specificity is low and only half of the patients have the confirmation of OA following proper testing [5,71].

### 4.2. Lung Function Tests

In order to diagnose OA, the presence of asthma and its relationship to the work environment must be confirmed. Asthma can be identified by the presence of reversible airflow obstruction (e.g., an increase of FEV_1_ greater than 12% and 200 mL after short-acting beta-2 agonist) [1]. Investigation for OA should be performed while the patient is still employed to compare at- and off-work lung function, to prove the variability and the reversibility [3,4]. However, a considerable number of workers investigated for OA have a normal lung function and data showed that pre-/post-shift monitoring of FEV_1_ were not sensitive or specific for OA [43,72,73].

Assessment of non-specific BHR is considered mandatory for the subjects without airflow limitation with suspicion of OA and can be performed by bronchial provocation with direct (e.g., methacholine/histamine) or indirect stimuli (e.g., exercise, mannitol, hypertonic saline, adenosine monophosphate). Methacholine and histamine inhalation challenges with a 20% fall in FEV_1_ are the most reliable and are well standardized [4]. A recently published review demonstrated that a negative methacholine challenge in a patient still exposed to the causative agent at work made the diagnosis of OA very unlikely [74]. It was also found that if the non-specific BHR was assessed shortly after a work shift and the result was negative, OA could be excluded [75]. However, if the worker has left the work environment for several days (a weekend may be enough) or weeks/months, the challenge test could be negative [76]. Despite the low specificity (36–40%), the presence of non-specific BHR to methacholine/histamine has a high sensitivity (87–95%) for the diagnosis of OA [53,74,77].

Monitoring the PEF increases the likelihood of a correct diagnosis. Malo et al. demonstrated that sensitivity and specificity of the PEF monitoring was optimal when PEF was measured every 2 hat- and off-work [78]. However, compared to the SIC, PEF monitoring has poor sensitivity and specificity. This is due to the fact that unlike FEV_1_, PEF cannot properly assess the airflow obstruction and also because collaboration of the worker is required. In some cases fear of losing a job or desire to get compensation could lead to false results. PEF and non-specific BHR are proven to be useful screening procedures when the worker is exposed to several sensitizers or when the offending agent is unknown [70].

SIC is considered to be the gold standard for confirmation of OA. It mimics the workplace exposure in a controlled environment [2]. SIC should be conducted in hospital-based specialised centers, by trained personnel and closely supervised by physicians with expertise in the field and able to manage acute asthmatic or anaphylactic reactions. SIC should be performed in enclosed challenge rooms equipped with an adequate exhaust ventilation system or using closed-circuit devices, with protective material for the patients and the technician. Contraindications for SIC include: uncontrolled asthma, FEV1 ≤60%, recent or unstable cardiovascular disease, uncontrolled epilepsy, pregnancy, recent (<4 weeks) respiratory tract infection, and a patient’s inability to understand the procedures. Controller asthma medication should be stopped according to their durations of action Tests should include a control challenge, a gradual increase of exposure to the suspected agent, and close monitoring of the patient during the challenge and for at least 6 h afterwards. According to the European consensus statement on SIC, a drop in FEV_1_ at least 15% from baseline for a minimum of 6 h after exposure to the suspected agent, is to be considered a positive response [79]. Because of potentially false positive or false negative responses to SIC and the needs of specialized facilities the test should be done in expert centers under close supervision, which explains the limited availability of SIC in the worldwide [2,79].

### 4.3. Immunological Workup and Molecular Diagnosis

SPT and assessments of sIgE represent effective ways to support the diagnosis of SI-OA IgE-mediated and can identify the offending agent for most HMW and some LMW agents (anhydride acids, platinum salts, reactive dyes, obeche wood) [5,80]. Immunological testing is not an applicable diagnostic approach for II-OA.

Unfortunately, standardized tests are available only for a few allergens and the allergen potency of SPT extracts may vary significantly among manufacturers [5,80], so the standardization of SPT for occupational allergens is a priority for the European Academy of Allergy and Clinical Immunology [81]. Negative SPT cannot entirely exclude the diagnosis of OA but makes it very unlikely [80]. Generally, the specificity and the positive predictive value (PPV) of SPT are low and a positive SPT could establish the diagnosis of sensitization to an occupational agent but cannot confirm the diagnosis of OA [5,81].

However, several studies showed that increasing the cutoff value for positive sIgE (e.g., ≥2.22 kU/L for wheat flour, ≥9.64 kU/L for rye flour, and ≥5.41 kU/L for latex) or for SPT responses (e.g., ≥3.5 mm to obeche wood dust, ≥5.0 mm for wheat flour, ≥4.5 mm for rye flour) significantly increased the specificity and PPV for OA [82,83,84,85].

Applying molecular diagnosis in OA can be helpful in identifying the allergens involved in OA and their cross reactivity with other occupational and non-occupational allergens. Molecular diagnosis was found to be useful in natural rubber latex allergy. For example, using the sum of sIgE for the recombinant allergens of *Hervea brasiliensis* (rHev b) 5 and 6.01 or 6.02 had a high PPV (>95%) for a positive SIC to latex with a high specificity (79%) [83,86]. Similarly, combining the presence of sIgE against some recombinant allergens of the wheat lipid transfer protein, *Triticum aestivum* (Tri a) 27, 28, 29.02, 32 and 39 showed a high specificity (97%) for wheat flour allergy among bakers [87].

BAT has been used in identifying several occupational allergens (e.g., obeche wood) and was superior to sIgE detection by ELISA [88]. A recent study demonstrated that BAT was able to discriminate sensitization from clinical allergy to latex in a small group of patients [89].

Although the immunologic assessment is interesting in OA, in practice there are important limits due to the lack of standardization and validation for most of the available extracts of occupational agents.

### 4.4. Biomarkers for the Assessment of Airway Inflammation

Biomarkers could increase the likelihood for a diagnosis of OA. According to a recent review the most accurate biomarkers for diagnosis and follow up are those associated with type 2 airway inflammation- sputum eosinophilia and FeNO [90].

A retrospective study demonstrated that elevated sputum eosinophilia (≥3%) at baseline had a high PPV for the diagnosis of OA caused by HMW and LMW agents [91]. Most subjects with SI-OA show an eosinophilic inflammatory response after SIC or exposure at work [5,80]. Using increasing cutoff values (e.g., >1%, >2% and >6.4%) for changes in sputum eosinophil percentage at work and off work, increased the specificity for the diagnosis of OA (76%, 80%, respectively 96%) [92]. An increase of sputum eosinophil counts >3% after SIC is a predictive factor for the occurrence of functional changes on subsequent exposures [91].

The use of FeNO in the diagnosis of OA is controversial [3]. Elevated FeNO levels were found in OA induced by HMW agents where an IgE-mediated mechanism was involved (e.g., baker’s asthma) as well as in OA induced by some LMW agents such as diisocyanates [93,94]. A cluster analysis demonstrated that FeNO levels were more consistently increased in patients with OA to HMW agents than in those to LMW agents [20]. A recent study found that an increase of FeNO  ≥  13 ppb following SIC is associated with a specificity of 90% for OA [95]. However, another study showed that a 2.2% increase in sputum eosinophilia had a greater sensitivity and PPV than a 10 ppb change in FeNO for a positive reaction to SIC [96]. A recent study found high serum periostin level in subjects with TDI-OA and suggested it as a potential biomarker for this phenotype of OA [97].

## 5. Management of Occupational Asthma

An early diagnosis is essential for a favorable outcome of the asthma. All patients with OA should be managed like other not work-related asthmatics with regard to asthma education, control of exposure to environmental triggers and appropriate pharmacotherapy. The pharmacologic therapy relies on a stepwise approach and is conducted according to the management guidelines. It is aimed to achieve good control of symptoms and minimize the future risk [1]. Workers who have had a high-level irritant exposure may require emergency treatment according to clinical practice recommendations [53]. However, even aggressive pharmacotherapy is never considered sufficient and should not be regarded as a reasonable alternative to environmental interventions.

### 5.1. Sensitizer-Induced Occupational Asthma

Ideally, patients with SI-OA should be completely relocated to a different occupation either in or outside the workplace [3]. A Cochrane review recently published evaluated the effectiveness of workplace interventions on OA and showed that complete removal from exposure, but not reduction of exposure, may improve lung function [98]. Conversely, a meta-analysis found that the reduction of exposure is associated with a lower likelihood of improvement (OR 0.16, 95%CI [0.03–0.91]) or recovery (OR 0.30, 95%CI [0.11–0.84]) of asthma symptoms, a higher risk of worsening of the symptoms (OR 10.23, 95%CI [2.97–35.28]) and non-specific BHR (OR 5.65, 95%CI [1.11–28.82]), compared with the complete avoidance of the exposure [99]. In addition, the workers with OA who remained exposed to the causal agent have a lower FEV_1_ than they completely removed outside the workplace [100]. However, viewing the substantial socio-economic impact, if relocation is not possible or not desired by the patient, interventions to decrease the exposure remain reasonable options despite a lower effectiveness on asthma outcomes than complete removal [3].

Primary, secondary and tertiary preventive measures could reduce the incidence and severity of SI-OA (Table 1). Occupational hygiene measures such as improvement of local ventilation and use of personal protective equipment could reduce the occupational exposure and the risk of OA (primary prevention). Several studies assessed the effectiveness of respiratory protective equipment and showed a significant reduction in respiratory symptoms and changes in pulmonary function for short-term exposures [101]. However, the use of respiratory protective equipment should not be regarded as a safe approach, especially in the long-term exposures and in patients with severe OA [102]. Worker education in the use of safe practices at work is mandatory in order to avoid deaths attributable to fatal exacerbations of OA already reported for diisocyanates and protein sensitizers [103].

Secondary prevention includes medical surveillance programs (e.g., medical questionnaires, spirometry, immunological testing) allowing early detections of OA in at-risk workers and the investigations, already detailed, to confirm the diagnosis [3,6].

Tertiary prevention involves the appropriate management after diagnosis with early removal of further exposure, pharmacological treatment, patient’s assistance with work-compensation claim and the monitoring of asthma control in future work environment [3,6]. Previous data identified several positive predictive factors for recovery after avoidance of exposure: younger age, shorter duration of contact, LMW agents’ exposure, early diagnosis and early removal. In contrast, older age, HMW agents’ exposure, lower lung function and longer duration of exposure at the time of the diagnosis had a negative role on the outcomes of OA [104]. However, only 32% of workers with OA reported recovery of asthma symptoms at 31 months after removal from the workplace [105]. Therefore, immediate cessation from work-related exposure to sensitizers should be strongly recommended to the worker and employer.

Usually, pharmacologic management follows clinical-practice guidelines for asthma. In individuals with IgE-mediated OA, allergen immunotherapy may be a useful option which potentially allows safe ongoing exposure to the workplace agent. A few immunotherapy studies have been carried out in health care workers who were allergic to latex but also in workers with wheat flour, sea squirt and rodents-induced OA [6]. Despite a benefice on respiratory symptoms, limitations to perform this treatment exist due to the lack of commercially validated extracts and the possibility to have systemic reactions [106]. On the other hand, biological treatments may represent a promising option in the management of selected cases of OA. Omalizumab, an anti-IgE antibody has shown to be an effective treatment for OA induced by HMW and LMW agents in atopic patients who remained exposed to the causal agent [107]. Obviously, biologics are upcoming while evolving therapies and further studies are needed.

### 5.2. Irritant-Induced OA

Unlike SI-OA, workers with II-OA may be able to continue the same work with appropriate asthma management and measures to prevent a further unintentional high-level exposure to irritants, without complete exposure elimination [3].

Following an acute inhalation of a high-level irritant agent, the rapid removal and protection of the workers from exposure are mandatory (e.g., use of respiratory protective equipment, evacuation of workplace, decontamination, eye wash). The management of acute II-OA include bronchodilator therapy, systemic corticosteroids and the administration of supplemental oxygen in the presence of hypoxemia [3]. With an adequate controller therapy, the workers who return to the same work environment should receive education regarding the effects of irritant exposures in asthma and have regular medical assessment including measurements of non-specific BHR. According to a position paper published recently, if uncontrolled asthma is developed at work, it requires complete removal from the workplace [53].

Obviously, the clinical and functional outcomes of II-OA seem to be remarkably similar to that in patients with SI-OA after cessation of exposure to the causative agent. Current data suggest a persistence of BHR several years after an acute inhalation accident [3]. A long term follow-up study showed that at the time of reassessment (mean interval 14 years) all patients still reported respiratory symptoms, non-specific BHR persisted in about three quarters of them and 68% of patients needed a controller medication for their asthma with inhaled corticosteroids [67].

High exposure to the inciting agent can be prevented through engineering controls, respiratory protective devices or job modification [108].

The optimal management of OA should include preventive measures, follow-up programs, medical evaluation, education of workers and treatment compliance (Table 2).

## 6. Conclusions

OA has become one of the most prevalent occupational lung diseases with a significant socio-economic impact on workers and society as a whole. OA is a heterogeneous disease with at least two phenotypes described according to the causative agent and the mechanisms involved in the pathogenesis: sensitizer-induced OA and irritant-induced OA.

The first step in the management of OA is to make a precise diagnosis with the identification of the causative agents in order to apply avoidance measures. The diagnosis is based on a history of occupational exposure and objective tests to confirm BHR related to workplace exposure. The most accurate is SIC, but other tests including the monitoring peak expiratory flow at- and off-work, assessment of non-specific BHR, sputum eosinophil count, FeNO measurement, and immunological testing which could give additional information for the diagnosis of OA. However, only a minority of work-related allergens are characterized on the molecular level and available for routine diagnosis. In addition, the access to SIC is limited to only a few expert centers and when the suspected agent has been identified. For these reasons, the diagnosis of OA is difficult and underestimated.

Once a sensitizer-induced OA is confirmed, early removal of the workers from exposure is associated with a better prognosis and outcomes. Complete avoidance is more effective than partial mitigation. Primary, secondary and tertiary prevention are mandatory in the management of sensitizer-induced OA. Patients with irritant-induced OA may continue to work if their respiratory symptoms are controlled with appropriate treatment. The pharmacologic therapy relies on a stepwise approach (similar to asthma management non-related to work), with the goal of obtaining and maintaining asthma control.

Recognizing asthma occupational triggers in the workplace, early diagnosis and removal of the workers from the exposure, education and development of institutional medical-surveillance programs for workers at risk could improve OA outcomes. In the other hand, a better standardization of diagnostic tests (allergens, biomarkers and SIC) and the identification of mechanisms driving different phenotypes of OA are needed and require further investigations.

## Figures and Tables

**Table 1:** Prevention of SI-OA.

Prevention	Measures
Primary	Avoidance of the introduction of new possibles sensitizing agents in the workplace Using safe alternatives to sensitizing agents Reduction of the sensitizing potential of agents by chemical or physical process Education programmes for workers to use safe practices at work Occupational hygiene measures to reduce exposure to work sensitizers (e.g., use of robotics, containment, ventilation) Monitoring and controlling exposure levels in the workplace
Secondary (early detection)	Institute medical-surveillance programs for workers at risk (e.g., periodic respiratory questionnaires, spirometry) Education of healthcare workers about OA Education of workers about the risk of OA and to recognize the symptoms of the disease (e.g., workplace or public education programs, information by healthcare provider)
Tertiary (appropiate treatment)	Evaluation of symptomatic workers to achieve an early and accurate diagnosis Workers’ relocation to reduce the risk of further exposure once the diagnosis is confirmed Controlling other possible triggers of asthma Pharmacological treatment to control asthma Patient’s assistance with work-compensation claim to limit socio-economic effects of the diagnosis Monitoring of the patient’s asthma control in future work environment to ensure safe placement.

Adapted from Cormier et al. [3] and Tarlo et al. [6].

**Table 2:** Management of OA.

Sensitizer-induced OA	Irritant-induced OA
- Cessation from work-related exposure to sensitizers - Consider reduction exposure - Allergen immunotherapy or biologics in selected cases	- Reduction of exposure
- Pharmacologic therapy - Follow-up programs (career change) - Medical evaluation - Education - Assist with compensation - Preventive measures	
